# Novel compound heterozygous variants of the *SEC23A* gene in a Chinese family with cranio-lenticulo-sutural dysplasia based on data from a large cohort of congenital cataract patients

**DOI:** 10.1186/s12920-023-01667-9

**Published:** 2023-10-12

**Authors:** Qiwei Wang, Xiaoshan Lin, Kunbei Lai, Yinghui Liu, Tingfeng Qin, Haowen Tan, Jing Li, Zhuoling Lin, Xulin Zhang, Xiaoyan Li, Haotian Lin, Weirong Chen

**Affiliations:** 1 State Key Laboratory of Ophthalmology, Zhongshan Ophthalmic Centre, Guangdong Provincial Key Laboratory of Ophthalmology and Visual Science, Guangdong Provincial Clinical Research Centre for Ocular Diseases, Sun Yat-sen University, 510060 Guangzhou, Guangdong Province China; 2 Department of Dermatology, Dermatology Hospital, Southern Medical University, 510091 Guangzhou, Guangdong Province China; 3 Aegicare Biotech, 518000 Shenzhen, Guangdong Province China

**Keywords:** *SEC23A*, Congenital cataract, Cranio-lenticulo-sutural dysplasia, Gene curation, Genetic variants

## Abstract

**Background:**

Cranio-lenticulo-sutural dysplasia (CLSD) is a rare dysmorphic syndrome characterized by skeletal dysmorphism, late-closing fontanels, and cataracts. CLSD is caused by mutations in the *SEC23A* gene (OMIM# 607812) and can be inherited in either an autosomal dominant or autosomal recessive pattern. To date, only four mutations have been reported to cause CLSD. This study aims to identify the disease-causing variants in a large cohort of congenital cataract patients, to expand the genotypic and phenotypic spectrum of CLSD, and to confirm the association between *SEC23A* and autosomal recessive CLSD (ARCLSD).

**Methods:**

We collected detailed medical records and performed comprehensive ocular examinations and whole-exome sequencing (WES) on 115 patients with congenital cataracts. After suspecting that a patient may have CLSD based on the sequencing results, we proceeded to conduct transmission electron microscopy (TEM) on the cultured skin fibroblasts. The clinical validity of the reported gene-disease relationships for the gene and the disease was evaluated using the ClinGen gene curation framework.

**Results:**

Two novel compound heterozygous variants (c.710A > C p.Asp237Ala, c.1946T > C p.Leu649Pro) of the *SEC23A* gene, classified as variant of uncertain significance, were identified in the proband with skeletal, cardiac, ocular, and hearing defects. The observation of typical distended endoplasmic reticulum cisternae further supported the diagnosis of CLSD. Application of the ClinGen gene curation framework confirmed the association between *SEC23A* and ARCLSD.

**Conclusion:**

This study expands the genotypic and phenotypic spectrum of CLSD, proposes TEM as a supplemental diagnostic method, and indicates that congenital cataracts are a typical sign of ARCLSD.

**Supplementary Information:**

The online version contains supplementary material available at 10.1186/s12920-023-01667-9.

## Introduction

Cranio-lenticulo-sutural dysplasia (CLSD, OMIM# 607812) is a dysmorphic syndrome characterized by skeletal dysmorphism, late-closing fontanels, and cataracts. The disease was initially described and named in an inbred Saudi Arabian family in 2003. It was subsequently linked to mutations in the *SEC23A* gene, which is located on chromosome 14q21.1 [1]. To date, only four mutations have been reported to cause CLSD (Table 1). A homozygous Phe382Leu substitution in the *SEC23A* gene was responsible for causing CLSD in the consanguineous Saudi Arabian family [2]. A heterozygous Met702Val substitution found in a boy with CLSD was inherited from his clinically unaffected father, suggesting the possibility of digenic inheritance [3]. A heterozygous Glu599Lys substitution was shared by a father and son with CLSD, indicating that the variant might have a dominant-negative effect [4]. Finally, a homozygous Met400Ile substitution in the *SEC23A* gene combined with an Arg334Cys substitution in the *MAN1B1* gene resulted in a disease phenotypically different from CLSD in a consanguineous family of Lebanese origin [5].

The *SEC23A* (OMIM# 610511) gene encodes the SEC23A protein, an essential component of the SEC23-SEC24 complex, part of the coat protein complex II (COPII), which plays a crucial role in vesicle budding during cargo molecules transport between organelles [6]. SEC 12 catalyzes the exchange of GDP-GTP on SAR1, activating the small GTPase SAR1, which, in turn, binds to the endoplasmic reticulum (ER) and recruits the Sect. 23-Sect. 24 complex to form the prebudding complex. SEC 24 contacts and captures the cargo molecules and then recruits the SEC13-SEC31 complex, inducing coat polymerization and membrane deformation. The vesicle with secretory proteins disassembles from the ER membrane after the conversion of SAR1-GTP to SAR1-GDP, ultimately transporting the cargo towards the Golgi complex [4, 5]. Skin fibroblasts from a patient harboring the *SEC23A* mutation exhibited a severe collagen secretion defect and distended ER, suggesting that the aberrant cargo molecule transport in the occurrence of CLSD [2].

Here, we identified two novel compound heterozygous variants of *SEC23A* in a Chinese girl born to non-consanguineous unaffected Han Chinese parents. The patient presented with typical features of CLSD, including developmental delay, frontal bossing, wide and delayed closure of the anterior fontanelle, bilateral congenital cataracts, and pectus excavatum. Compared with her unaffected parents with heterozygous *SEC23A* variant, the ER from the patient’s fibroblasts was distended as seen with transmission electron microscopy (TEM). Our finding broadens the genotypic and phenotypic spectrum of CLSD via detailed examinations and improves the precision of genetic counseling.

**Table 1:** Clinical characteristics of the CLSD patients

ID	cDNA Change	Protein Change	Frequency of the variants in gnomAD	Inheritance Pattern	Gene inheritance Pattern	Ocular abnormality	Systemic abnormality
1	c.1144T>C	p.Phe382Leu	NA	Autosomal recessive	Homozygous	Hypertelorism, prominent supraorbital ridge, downslanting palpebral fissures, Y-shaped sutural cataracts	Short stature, wide open calvarial sutures with large and late-closing anterior fontanelles, abnormal hair, frontal bossing, hyperpigmentation with capillary hemangioma of the forehead, mid-face hypoplasia, broad and prominent nose, dental abnormalities, long smooth philtrum, and wide mouth with thin upper lip
2*	c.1200G>C	p.Met400Ile	NA	Autosomal recessive	Homozygous	Hypertelorism, long palpebral fissures, and ptosis	Moderate global developmental delay, tall stature, obesity, macrocephaly, mild dysmorphic features, hypertelorism, maloccluded teeth, intellectual disability, and flat feet
3	c.1795G>A	p.Glu599Lys	NA	Autosomal dominant	Heterozygous	Hypertelorism and bilateral glaucoma with exfoliation of the lens capsule	Large fontanelle with wide cranial sutures, large forehead, thin nose, high arched palate, and micrognathia
4**	c.2104 A>G	p.Met702Val	3.22e-4	Autosomal dominant	Heterozygous	Hypertelorism, bilateral exotropia,bilateral optic atrophy, and a double-ring sign	Large anterior fontanelle, valvular pulmonic stenosis, and motor delay
5	c.710 A>C c.1946T>C	p.Asp237Ala p.Leu649Pro	NA NA	Autosomal recessive	Compound heterozygous	Hypertelorism, nystagmus, and bilateral congenital total cataracts	Large anterior fontanelle, frontal bossing, abnormal hair, patent foramen ovale, wide nasal bridge, long philtrum, thin vermilion border, pectus excavatum, and developmental retardation

NA: not available; *The patient also carried a homozygous Arg334Cys substitution in the *MAN1B1* gene; ** The heterozygous Met702Val substitution found in the patient was inherited from his clinically unaffected father

## Methods

### Patient enrollment and clinical assessments

This study adhered to a protocol approved by the Institutional Review Board of the Zhongshan Ophthalmic Center, Sun Yat-Sen University, following the Declaration of Helsinki. A total of 115 patients with congenital cataracts were included in the present study which is a part of the Childhood Cataract Program of the Chinese Ministry of Health (CCPMOH) [7]. The parents of the probands gave written informed consent before participating in the study. The clinical records of the proband were collected. Thorough ocular examinations were performed using slit-lamp photography (BX900; Haag-Streit, Bern, Switzerland), the Pentacam scheimpflug system (Oculus, Wetzlar, Germany), Tono-Pen (Reichert, Depew, NY, USA), A- and B-scan ultrasound (Aviso, Quantel Médical, Clermont-Ferrand, France), RetCam (Natus Medical Incorporated, Pleasanton, CA, USA), and optical coherence tomography (OCT; Cirrus HD-OCT 5000, Carl Zeiss Meditec, Dublin, CA, USA).

### Variant screening and pathogenicity analysis

Genomic DNA from the probands and their available parents was extracted from peripheral leukocytes using the QIAamp DNA Mini Kit (QIAGEN, Hilden, Germany) in accordance with the manufacturer’s instructions. WES was performed on all probands using Illumina next-generation sequencing systems (Illumina, San Diego, CA, USA) [8]. The raw reads were mapped to the human reference genome (hg19/GRCh37) and a bioinformatic assessment was performed as described previously [8, 9]. Copy number variants and sub-microscopic deletions/duplications were analyzed using CNVkit [10]. The existence of *SEC23A* variants (NM_006364.4) was verified in the proband and her parents by Sanger sequencing. The pathogenicity of the compound heterozygous variants was assessed following the American College of Medical Genetics and Genomics/Association for Molecular Pathology variant (ACMG/AMP) classification guidelines published in 2015 [11–14].

### TEM

Skin biopsies of the proband and her parents were used to generate fibroblasts using standard conditions [15]. The fibroblasts were cultured in Dulbecco’s modified Eagle’s medium containing 10% fetal bovine serum to 70% confluence before harvest. They were fixed with 1% OsO4 in 0.1 M phosphate buffer (pH 7.4), dehydrated at room temperature, resin penetrated and embedded, and then polymerized. The samples were sectioned on an ultramicrotome (Leica EM UC7), and stained in cuprum grids with 2% uranium acetate saturated alcohol solution and 2.6% lead citrate. The sections were observed by TEM (Hitachi HT-7800, Japan). The ER cisternae larger than 150 nm were considered distended [3].

### *SEC23A* gene curation

The association of the *SEC23A* gene and autosomal recessive CLSD (ARCLSD) was classified using the ClinGen gene curation framework (ver. 9) following standard procedures (https://clinicalgenome.org/docs/gene-disease-validity-standard-operating-procedure-version-9/). A semiquantitative score was calculated based on the genetic and experimental evidence extracted from the literature to evaluate the strength of the evidence, which was discussed by experts (including HT, HL, and WC). The association was graded as definitive (12–18 points AND replication over time), strong (12–18 points), moderate (7–11 points), limited (0.1–6 points), no evidence, disputed, or refuted [9, 16].

## Results

### Molecular findings

**Fig. 1:**
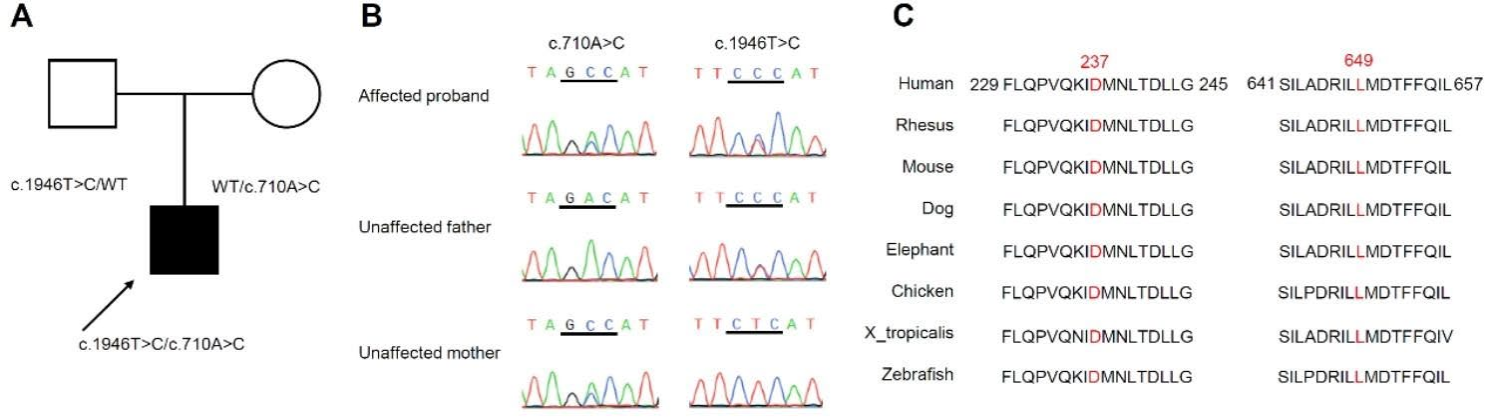
Molecular findings of the Chinese Han CLSD family. **(A)** The pedigree. **(B)** Sanger sequencing chromatograms of the patient and her unaffected parents. **(C)** Comparison and alignment of the *SEC23A* protein sequences of eight species

**Fig. 2:**
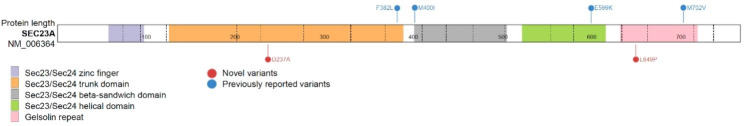
The spectrum of our (red dots) and reported (blue dots) *SEC23A* variants

As a part of the CCPMOH etiology detection program, 115 patients with congenital cataracts underwent WES. Two novel heterozygous *SEC23A* variants (Fig. 1) affecting two highly conserved amino acids were found in the girl born to non-consanguineous unaffected Han Chinese parents. The c.710 A > C variant (maternal origin) changes the conserved 237 aspartic acid into alanine (p.Asp237Ala) in the Sec23/Sec24 trunk domain, and the c.1946T > C variant (paternal origin) changes the conserved 649 leucine into proline (p.Leu649Pro) in the Gelsolin repeat domain (Fig. 2). The c.710 A > C variant was classified as variant of uncertain significance (VUS) showing evidence of PM2_support + PP3 + PP4_moderate. The c.1946T > C variant was also classified as VUS showing evidence of PM2_support + PP3_moderate + PP4_moderate.

### Clinical manifestations

**Fig. 3:**
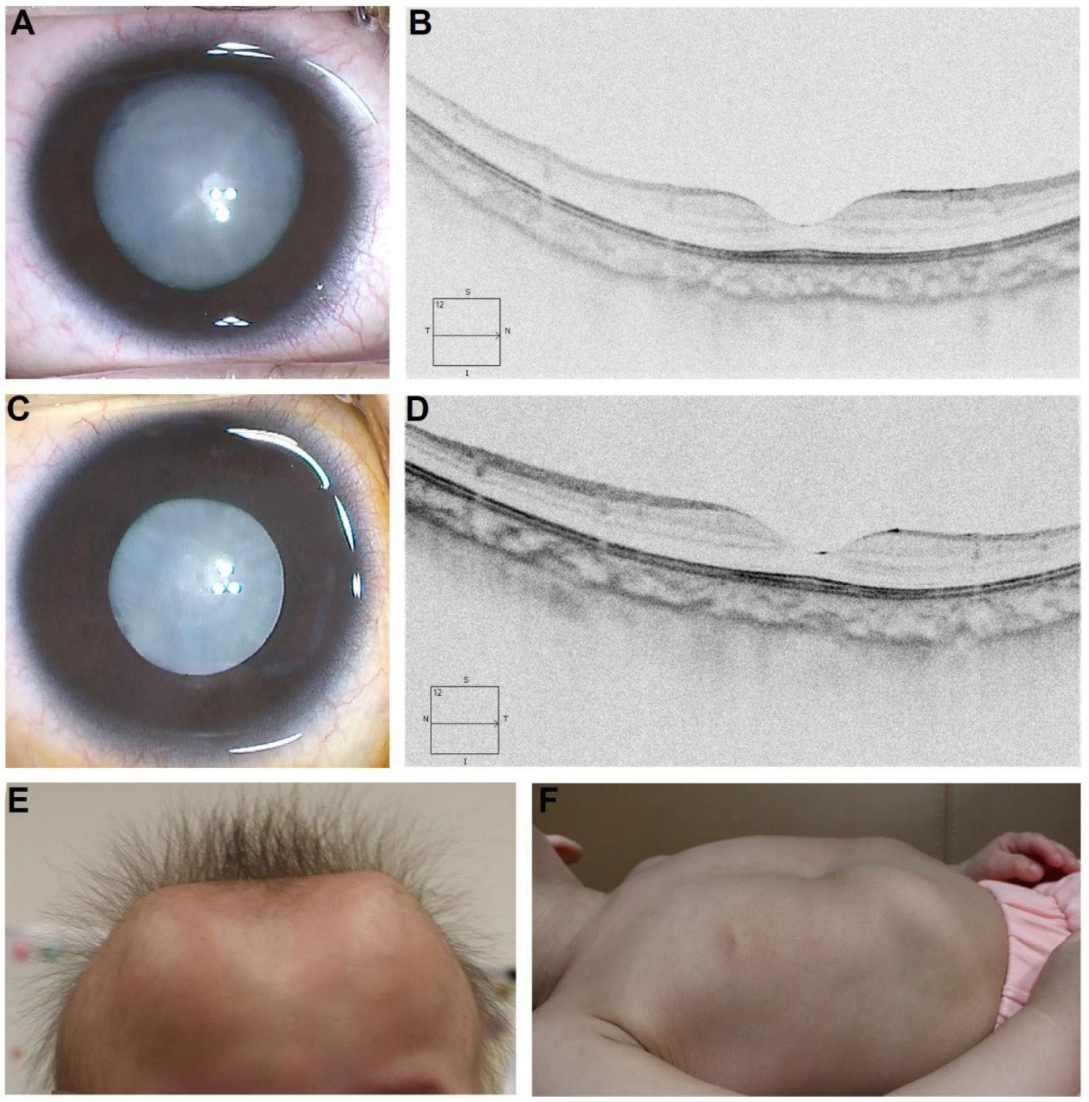
The ocular and craniofacial features of a CLSD patient. Bilateral total cataracts and normal macular (**A** to **D**). OCT revealed that the central fovea structure of the macular was clearly visible. Besides, the thickness of each layer was within the normal limits. Frontal bossing, wide and delayed closure of the anterior fontanelle, sparse and coarse hair, and pectus excavatum (**E** and **F**)

Prenatal ultrasound had suggested intrauterine growth retardation of the girl during her mother’s pregnancy. At birth, she was found to have whitish pupils, a large anterior fontanelle with a 31 cm head circumference, patent foramen ovale, and bilateral otoacoustic emission test failure. Physical and ocular examinations revealed frontal bossing, wide delayed closure of the anterior fontanelle, sparse and coarse hair, hypertelorism, nystagmus, bilateral congenital total cataracts, wide nasal bridge, long philtrum, thin vermilion border, and pectus excavatum (Fig. 3). The patient was brought to the ophthalmic clinic at the age of 20 months and underwent bilateral cataract extractions shortly thereafter. Postoperative ocular examination showed that the fundus of both eyes were normal. The physical examination 2 years postoperatively indicated developmental retardation. The girl was still unable to walk or talk when she was 32 months old. The bilateral best corrected visual acuity was 0.04 with normal intraocular pressure. Twice-yearly ocular and pediatric follow-ups were scheduled.

### Electron microscopy micrographs

**Fig. 4:**
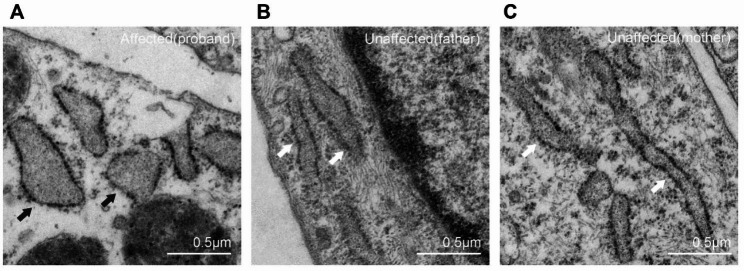
Electron microscopy micrographs of cultured fibroblasts. Fibroblasts from the patient carrying compound heterozygous *SEC23A* gene variants had dilated ER (black arrows) **(A)**. The unaffected father **(B)** and mother **(C)** carrying heterozygous *SEC23A* variants had normal endoplasmic reticulum (white arrows)

The fibroblasts of the proband and her parents were examined by thin-section electron microscopy. Cells from the unaffected father carrying the heterozygous c.1946T > C variant and the unaffected mother carrying the heterozygous c.710 A > C variant showed typical morphology of the rough ER with narrow cisternae. By contrast, most of the cells of the patient carrying compound heterozygous variants had distended ER cisternae (Fig. 4).

### *SEC23A* gene curation

The gene curation of *SEC23A*-ARCLSD was moderate (Table S1) for the following reasons. First, both identified variants were missense variants inherited from consanguineous parents, with functional data derived from the patient’s fibroblasts [2, 5]. Second, a multipoint linkage analysis of a large Saudi Arabian family resulted in a 5.44 maximum LOD score. Therefore, 3 points are allotted for segregation evidence [1, 2]. The expression data provide functional evidence that associates *SEC23A* with CLSD. The gene was expressed ubiquitously consisting of the typical multisystemic abnormalities of the disease. The distinct CLSD phenotype was observed in sec23a-blocking morpholino zebrafish embryos and a *Sec23a* deficient mice model [2, 17] contributing 4 points. Therefore, a total of 8.5 points are given to *SEC23A*-ARCLSD. The calculated classification is of moderate.

## Discussion

We identified two novel compound heterozygous *SEC23A* gene variants in a Chinese girl exhibiting characteristic clinical manifestations of CLSD. TEM observations of cultured skin fibroblasts showed typical distended ER cisternae, providing additional diagnostic evidence for CLSD. This study not only expands the genotypic and phenotypic spectrum of the *SEC23A* gene but also highlights congenital cataracts as a typical sign of ARCLSD.

The presence of typical distended ER cisternae, as detected by TEM in cultured skin fibroblasts, serves as supplementary diagnostic evidence for CLSD. Despite both variants being predicted to be highly deleterious by several bioinformatics analysis programs, they were classified as VUS, which is insufficient for diagnosis, prenatal intervention, or termination [18]. Consequently, we performed skin biopsies on the proband and her parents to generate fibroblasts. Thin-section electron microscopy of these culture cells revealed abnormally distended ER cisternae in the patient and typical narrow ER cisternae in her unaffected parents. These findings underscore the importance of TEM as a supplementary diagnostic tool for CLSD and a guide for clinical decision-making.

Congenital cataracts present a characteristic clinical manifestation of ARCLSD. Patients carrying either the homozygous variant (c.1144T > C) or compound heterozygous variants (c.710 A > C and c.1946T > C) in *SEC23A* present with congenital cataracts. In contrast, congenital cataract is absent in patients carrying a heterozygous variant of *SEC23A* (c.2104 A > G or c.1795G > A). Congenital cataract occurs result from the opacity of the lens and can be easily detected through the transparent cornea and pupil. Therefore, they often serve as the initial sign of syndromic disease in infants. The examination for congenital cataract helps predict genetic patterns and inform genetic screening and counseling for patients with skeletal defects suspected of having CLSD.

The ClinGen gene curation of associations between the *SEC23A* gene and ARCLSD is of great importance. It classifies the association of a gene with a disease according to a semiquantitative score, calculated based on genetic and experimental evidence extracted from the literature [19]. The establishment of gene–disease associations is the foundation for subsequent ACMG analysis, which provides standards and guidelines for interpreting variants of a specific gene. Therefore, understanding the strength of the gene–disease association can facilitate making a definitive diagnosis and providing guidance for genetic counseling and clinical decision-making [14, 20]. In this study, the association between the *SEC23A* gene and ARCLSD was curated and classified as moderate according to the ClinGen Lumping and Splitting guidelines. While there have only been two reported pedigrees so far, as more variants are reported over time, the evidence supporting this association is expected to become strong/definitive.

This study has limitations as we only present data from a single patient. CLSD is an extremely rare disease with only four reported cases so far. Despite including clinical data from over 4400 congenital cataract patients and conducting WES on more than 100 congenital cataract patients in our clinical database (NCT01417819) [7], we only identified one case. The identified variants were classified as VUS due to insufficient evidence. Therefore, we performed further TEM on fibroblasts from the participants and proposed typical dilated ER as supplementary evidence for the diagnosis of CLSD. We will continue to collect more cases and evidence related to CLSD to further our understanding of the disease.

## Conclusions

In conclusion, we have identified two compound heterozygous *SEC23A* variants in a patient presenting with skeletal, cardiac, ocular, and hearing abnormalities. By integrating thespecific clinical features, fibroblast changes, and genetic screening outcomes, CLSD was diagnosed. Further evidence-based curations verified the association of *SEC23A* and ARCLSD. Our findings expand the genotypic and phenotypic spectrum, propose TEM as a additional diagnostic method, and highlight congenital cataracts as a distinct sign of ARCLSD.

### Electronic supplementary material

Below is the link to the electronic supplementary material.

Supplementary Material 1: Additional file 1: Table S1

## Data Availability

To protect the security of human genetic resources, this study does not deposit the raw data in publicly available repositories. The data is available from the corresponding authors upon reasonable request.
