# Supplementary material for: Novel compound heterozygous variants of the SEC23A gene in a Chinese family with cranio-lenticulo-sutural dysplasia based on data from a large cohort of congenital cataract patients
Source: BMC Med Genomics. 2023 Oct 12;16:241. doi: 10.1186/s12920-023-01667-9 (PMC10568747; doi:10.1186/s12920-023-01667-9)
Supplement: Supplementary file 1 — Supplementary Material 1: Additional file 1: Table S1 [file 12920_2023_1667_MOESM1_ESM.docx]

**Supplementary Table S1.** Genetic and experimental evidence summary matrix about *SEC23A* and autosomal recessive CLSD (OMIM #607812)
